# Unambiguous Stereochemical Assignment of Cyclo(Phe-Pro), Cyclo(Leu-Pro), and Cyclo(Val-Pro) by Electronic Circular Dichroic Spectroscopy

**DOI:** 10.3390/molecules26195981

**Published:** 2021-10-02

**Authors:** Alison Domzalski, Liliana Margent, Valeria Vigo, Faizunnahar Dewan, Naga Vara Kishore Pilarsetty, Yujia Xu, Akira Kawamura

**Affiliations:** 1 Biochemistry Ph.D. Program, The Graduate Center of CUNY, New York, NY 10016, USA; adomzalski@gradcenter.cuny.edu (A.D.); fdewan@gradcenter.cuny.edu (F.D.); yxu0001@hunter.cuny.edu (Y.X.); 2 Department of Chemistry, Hunter College of CUNY, New York, NY 10065, USA; LILIANA.MARGENT54@myhunter.cuny.edu (L.M.); VALERIA.VIGO71@myhunter.cuny.edu (V.V.); 3 Department of Radiology, Memorial Sloan Kettering Cancer Center, New York, NY 10065, USA; pillarsn@MSKCC.ORG; 4 Chemistry Ph.D. Program, The Graduate Center of CUNY, New York, NY 10016, USA

**Keywords:** diketopiperazines, epimerization, mixed microbial culture, stereochemistry, electronic circular dichroism, biofilms

## Abstract

2,5-diketopiperazines (DKPs) are cyclic dipeptides ubiquitously found in nature. In particular, cyclo(Phe-Pro), cyclo(Leu-Pro), and cyclo(Val-Pro) are frequently detected in many microbial cultures. Each of these DKPs has four possible stereoisomers due to the presence of two chirality centers. However, absolute configurations of natural DKPs are often ambiguous due to the lack of a simple, sensitive, and reproducible method for stereochemical assignment. This is an important problem because stereochemistry is a key determinant of biological activity. Here, we report a synthetic DKP library containing all stereoisomers of cyclo(Phe-Pro), cyclo(Leu-Pro), and cyclo(Val-Pro). The library was subjected to spectroscopic characterization using mass spectrometry, NMR, and electronic circular dichroism (ECD). It turned out that ECD can clearly differentiate DKP stereoisomers. Thus, our ECD dataset can serve as a reference for unambiguous stereochemical assignment of cyclo(Phe-Pro), cyclo(Leu-Pro), and cyclo(Val-Pro) samples from natural sources. The DKP library was also subjected to a biological screening using assays for *E. coli* growth and biofilm formation, which revealed distinct biological effects of cyclo(D-Phe-L-Pro).

## 1. Introduction

2,5-diketopiperazines (DKPs) are cyclic dipeptides isolated from various natural sources [1]. Each DKP has four possible stereoisomers, except those containing glycine. Since their chirality centers are on the rigid core framework [2], each stereoisomer projects sidechains in unique spatial orientations, which could result in distinct biological effects. In fact, DKPs are known for a wide variety of biological properties, including anti-biofilm, antibiotic, antiviral, anticancer, and neuroprotective properties [3,4,5]. Characterization of the relationship between DKP stereochemistry and biological effects, however, has been hampered by the lack of a reliable method to determine the absolute configuration of DKP samples. 

At present, the standard methods for stereochemical assignment of DKPs are optical rotation and Marfey’s method. However, these methods have some limitations, which make them unsuitable for stereochemical characterization of DKPs from natural sources. Optical rotation is subject to high variability depending on concentration and solvent [6]. Furthermore, optical rotation typically requires a large amount (>10 mg) of purified sample [7], which is often difficult to secure from natural sources. On the other hand, Marfey’s method [8] requires acid hydrolysis of DKP to generate amino acid constituents, which are subsequently derivatized and subjected to HPLC analysis to determine stereochemistry. Acid hydrolysis, however, is problematic because DKPs are prone to epimerization in both acidic and basic environments [9]. The use of these methods has contributed to conflicting stereochemical assignments of DKPs in the literature. For example, stereochemistry of DKPs from the marine sponge *Calyx* CF. *podatypa* [10], which had been used as the basis for stereochemical assignments of DKPs from other sources [3,11], turned out to be incorrect [12]. 

During our recent study on mixed microbial cultures (MMCs), which were derived from microbiotas of plants and environmental samples [13], we analyzed our datasets of liquid chromatography tandem mass spectrometry (LC/MS/MS) with GNPS (Global Natural Products Social Molecular Networking), which is a web-based MS database, to characterize metabolites in complex mixtures [14]. The GNPS analysis revealed a series of DKPs, namely cyclo(Phe-Pro), cyclo(Leu-Pro), and cyclo(Val-Pro), in MMCs (Supplementary Figure S1). The identification of these DKPs underscored the need for a simple, sensitive, and reproducible method for the stereochemical assignment of these DKPs. To address this need, we decided to explore the utility of electronic circular dichroic (ECD) spectroscopy. 

ECD has clear advantages over optical rotation and Marfey’s method. It requires much less sample than optical rotation. In addition, unlike Marfey’s method, DKP samples can be directly subjected to ECD measurements without acid hydrolysis. Although ECD has been used for conformational analysis of some DKPs [15], it remains to be determined whether ECD can differentiate all four stereoisomers of each DKP. 

Here, we first present the synthesis of all stereoisomers of cyclo(Phe-Pro), cyclo(Leu-Pro), and cyclo(Val-Pro), and spectroscopic characterization of synthesized compounds by MS, NMR, UV, and ECD. The key question to be addressed in this study is whether the resulting ECD spectral dataset can serve as a reference to unambiguously determine the absolute configuration of unknown DKP samples. In addition, we also present our preliminary biological study of what may be called the “stereochemistry–activity relationship” of DKPs using *E. coli* growth and biofilm assays. 

## 2. Results

### 2.1. Synthesis of all Stereoisomers of Cyclo(Phe-Pro), Cyclo(Leu-Pro), and Cyclo(Val-Pro) 

**Scheme 1:**
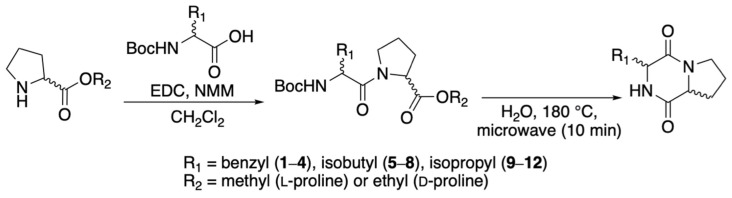
Synthesis of DKP stereoisomers [16].

**Figure 1:**
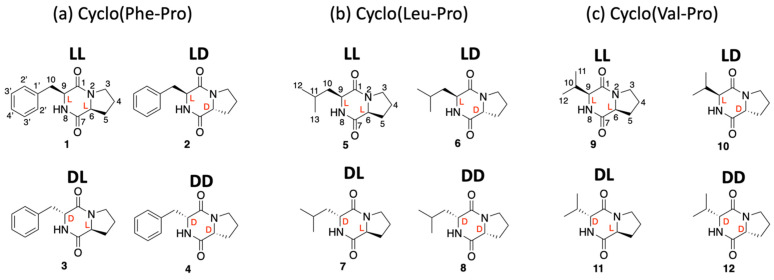
Synthetic library of 2,5-diketopiperazines containing all possible stereoisomers of (**a**) cyclo(Phe-Pro) (**1**–**4**), (**b**) cyclo(Leu-Pro) (**5**–**8**), and (**c**) cyclo(Val-Pro) (**9**–**12**).

DKP stereoisomers were synthesized by using a scheme developed by Tullberg et al. (Scheme 1) [16]. The crude product of each synthesis was purified by semi-preparative HPLC and subjected to spectroscopic characterization by MS and NMR. Figure 1 shows the library of 12 compounds synthesized for the ECD analysis and biological studies (Figure 1). Table 1 shows the molar absorptivity (*ε*-value) of each stereoisomer in methanol (spectroscopic grade), which can be used to quantify samples of cyclo(Phe-Pro), cyclo(Leu-Pro), and cyclo(Val-Pro) purified from natural sources. 

### 2.2. Differentiation of DKP Stereoisomers by ECD

**Figure 2:**
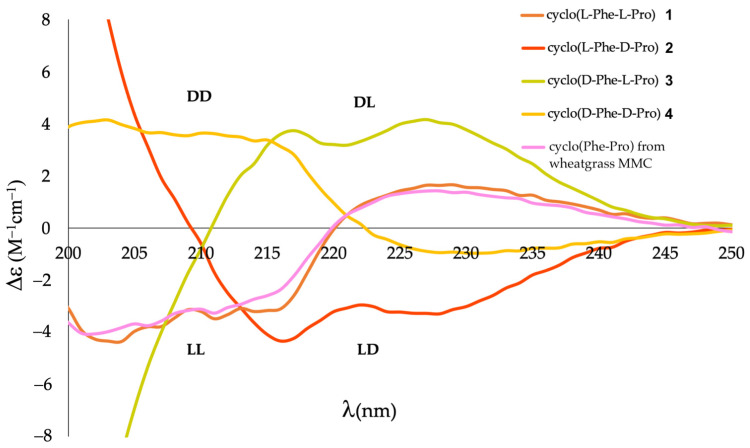
ECD spectra of synthetic cyclo(Phe-Pro) stereoisomers and a cyclo(Phe-Pro) sample with unknown stereochemistry from wheatgrass MMC (pink). Methanol (spectroscopic grade) was used as the solvent.

**Figure 3:**
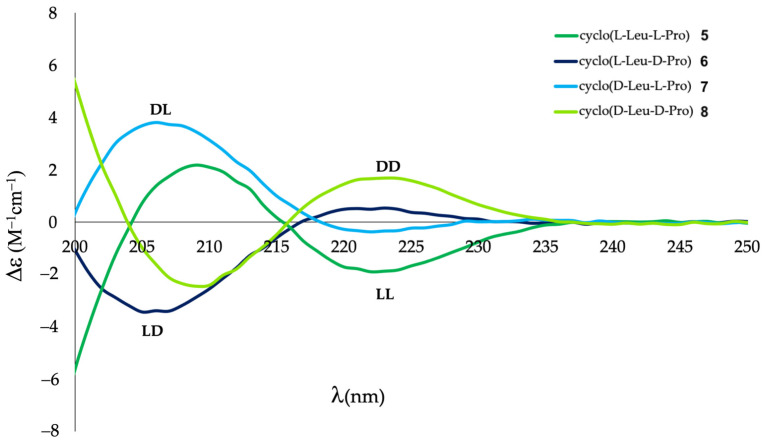
ECD spectra of synthetic cyclo(Leu-Pro) isomers in methanol (spectroscopic grade).

**Figure 4:**
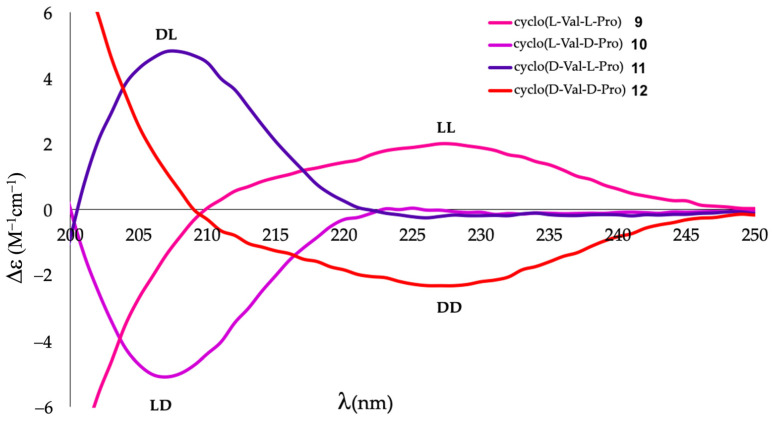
ECD spectra of synthetic cyclo(Val-Pro) isomers in methanol (spectroscopic grade).

In order to determine the ability of ECD to differentiate DKP stereoisomers, the synthetic compounds were subjected to ECD measurements. Gratifyingly, the signal in the 200–250 nm region was strong enough to produce an informative spectrum for each DKP examined. As shown in Figure 2, all four stereoisomers of cyclo(Phe-Pro) were readily differentiated by ECD, which revealed symmetrical spectra for each enantiomeric pair (LL/DD and LD/DL). Likewise, stereoisomers of cyclo(Leu-Pro) and cyclo(Val-Pro) could also be readily distinguished by ECD (Figure 3 and Figure 4, respectively). It is noted that ECD can be measured reproducibly at ~0.1 mg/mL, which enables microscale stereochemical characterization of DKP samples. 

### 2.3. Stereochemical Assignment of Cyclo(Phe-Pro) from Wheatgrass MMC

Having prepared the reference ECD spectra of cyclo(Phe-Pro), cyclo(Leu-Pro), and cyclo(Val-Pro) stereoisomers, we decided to characterize the stereochemistry of DKPs that were previously detected in MMCs by the GNPS analysis (Supplementary Figure S1). A large-scale sample preparation was carried out from 30 L of wheatgrass MMC. While we could not obtain much cyclo(Leu-Pro) and cyclo(Val-Pro), the amount of purified cyclo(Phe-Pro) was sufficient (0.3 mg) for stereochemical analysis. MS and ^1^H NMR indicated that the purified sample was either cyclo(L-Phe-L-Pro) (**1**) or cyclo(D-Phe-D-Pro) (**4**). The subsequent ECD measurement unambiguously determined its stereochemistry as cyclo(L-Phe-L-Pro) (**1**) (Figure 2, the pink spectrum). 

### 2.4. Stereochemistry–Activity Relationship of DKPs

While DKPs are known for their diverse biological effects [3,4,5], it is largely unknown how their stereochemistry might impact their biological activity. Our library of synthetic DKPs with defined stereochemistry creates an opportunity to characterize their stereochemistry–activity relationship, which would ultimately enable us to understand the structural basis of their diverse biological effects. As a first step toward this goal, we examined the effects of DKP stereoisomers on *E. coli* growth and biofilm formation. 

**Figure 5:**
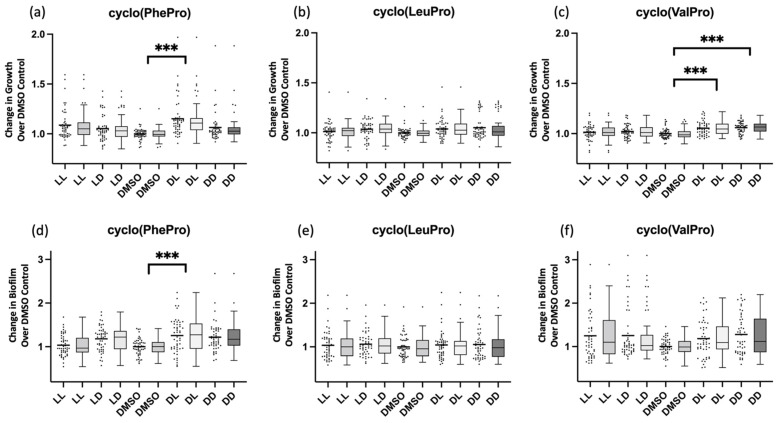
Effects of the synthetic DKP stereoisomers on *E. coli* growth (**a**–**c**) and biofilm formation (**d**–**f**). Both scatter and box-and-whisker plots are presented for each stereoisomer. The effects are normalized by the DMSO control. *E. coli* cells were treated with the samples (0.1 mg/mL, n = 50) and incubated at 37 °C for 24 h. Overall growth of *E. coli* was estimated by the OD_600_ measurement after incubation. Biofilm formation was determined by the crystal violet assay. The horizontal line in the scatter plot represents the mean, whereas the middle line in the box-and-whisker plot is the median. DMSO: vehicle control. *** *p* < 0.0001 (*t*-test).

Figure 5 shows the growth and biofilm formation of *E. coli* treated with different DKP stereoisomers. The effects of DKPs are normalized by a DMSO control. Since the effects of DKPs on *E. coli* were overall modest, we examined 50 replicates for each stereoisomer and focused our analysis on stereoisomers with the most prominent effects (Student’s *t*-test *p*-value of 0.0001 or lower). The result of each stereoisomer is presented in both scatter and box-and-whisker plots to show the distribution of 50 replicates. The study revealed that cyclo(D-Phe-L-Pro) promotes both *E. coli* growth and biofilm formation significantly (*p*-values = 1.3 × 10^−6^ and 9.3 × 10^−5^, respectively). Cyclo(D-Val-L-Pro) and Cyclo(D-Val-D-Pro) also promoted *E. coli* growth (*p* = 3.0 × 10^−5^ and 3.0 × 10^−7^, respectively). On the other hand, none of the cyclo(Leu-Pro) stereoisomers displayed a significant effect. 

## 3. Discussion 

DKPs are ubiquitously found in microbial communities in humans, animals, plants, and the environment. They are believed to play important roles in microbial communication [4,17,18,19]. From the standpoint of biosynthesis, it makes sense for microbes to use DKPs as chemical messengers. The building blocks, i.e., amino acids, are readily available. Permutations of amino acids make it possible to rapidly generate structurally diverse DKPs. Some microbes are equipped with epimerases, which can further diversify their structures. The diverse structures, in turn, translate into different biological effects, thereby enabling microbes to fine-tune their messages. Thus, in order to understand chemical communication mediated by DKPs, precise characterization of their structures, including stereochemistry, is essential. At present, however, most studies on DKPs stop short of stereochemical assignment. Since DKPs have been studied extensively for many decades [1,4,5], there is the perception of DKPs as a “known” class of compounds, which might discourage detailed structural characterization. Stereochemical characterization of DKPs, however, is far from over. The literature is fraught with misinformation about their stereochemistry, which has been perpetuated by the reports of incorrect stereochemical assignments and repeated citations of those reports. The situation has also been exacerbated by the continued use of inadequate methods for stereochemical characterization. Clearly, there remains much more work to be carried out before we understand the roles of DKP stereochemistry in microbial communication.

The current study demonstrates the utility of ECD for stereochemical characterization of cyclo(Phe-Pro), cyclo(Leu-Pro), and cyclo(Val-Pro), which are detected in various microbial communities. Stereoisomers of these DKPs, **1–12**, exhibit distinct ECD spectra (Figure 3, Figure 4 and Figure 5). As such, when a new sample of these DKPs, as determined by MS and NMR^1^, is purified from a microbial community, its stereochemistry can be readily assigned by ECD measurement, followed by comparison with the reference spectra of **1–12**. In fact, we successfully determined the stereochemistry of cyclo(L-Phe-L-Pro) from wheatgrass MMC with a sub-milligram quantity (0.3 mg) of the purified material. It is our expectation that this ECD-based approach will facilitate the characterization of the occurrence of **1–12** in various microbial communities. 

While the current study obtained reference ECD spectra by using synthetic DKPs, it is also possible to obtain reference spectra through theoretical calculation [20]. The theoretical approach is important, especially for DKPs that are not readily obtained by synthesis. In fact, theoretical ECD has recently become an important approach for stereochemical analysis of structurally complex DKPs from natural sources [21,22]. Another possible alternative approach is the use of vibrational CD (VCD), which has become a powerful method for determining absolute configuration of natural products [23]. Although VCD requires more sample materials than ECD, VCD provides a wealth of structural information associated with individual functional groups, which could be useful for differentiation of complex DKP stereoisomers. In fact, VCD has been used for stereochemical assignment of cyclic peptides, including DKP [24,25,26]. 

The synthesis of DKP stereoisomers, **1–12**, has also created an opportunity to systematically study their stereochemistry–activity relationship using in vitro assays, such as the biofilm assay. Although there have been reports of the biofilm modulation by DKPs, they either only identified the constituent amino acids present without attending to stereochemistry, or only looked at one stereoisomer without comparison to others [3,19,27,28,29]. The current study, on the other hand, is the first to screen all stereoisomers of cyclo(Phe-Pro), cyclo(Leu-Pro), and cyclo(Val-Pro), which revealed the most pronounced effects of cyclo(D-Phe-L-Pro) on both *E. coli* growth and biofilm formation. Cyclo(D-Val-L-Pro) and cyclo(D-Val-D-Pro) also increased, albeit modestly, the *E. coli* growth. These findings are reminiscent of a study by Fdhila et al. [3], which indicated the importance of at least one D-amino acid in the DKP framework for the growth inhibition of *Vibrio anguillarum*. Further studies with other assays are on-going to obtain a comprehensive stereochemistry–activity relationship. 

## 4. Materials and Methods

### 4.1. General Experimental Procedures

ECD spectra were obtained on an Applied Photophysics (Leatherhead, UK) Chirascan™ V100 Circular Dichroism Spectrophotometer with a 1 mm optical path quartz cuvette. Spectra were obtained at 25 °C at a wavelength range from 190 to 300 nm at a concentration of 0.1 mg/mL in methanol (spectroscopic grade). Ellipticity measurements were corrected against a methanol background using the same cuvette. CD data were analyzed using the Chirascan software. UV spectra were collected on a Beckman-Coulter (Miami, FL, USA) DU^®^ 800 UV/Visible Spectrophotometer with a 1 cm optical path length quartz cuvette. Spectra were measured at room temperature at a wavelength range from 190 to 300 nm. Molar absorptivity measurements were corrected against a methanol background using the same cuvette. One- and two-dimensional NMR spectra were recorded on 600 MHz Avance III, 500 MHz Avance DRX, and NEO-500 NMR spectrometers by Bruker (Carteret, NJ, USA) using CDCl_3_ as the solvent. TMS was used as a reference. Positive ion MS spectra were obtained using both an Agilent (Santa Clara, CA, USA) iFunnel 6550 Q-ToF LC/MS system using m/z 120.0808 and 922.0098 as reference masses and an Agilent 6340 Ion Trap with an Electron Transfer Dissociation LC/MS system. Collision energy for HRESI-MS/MS settings was 35 eV. For column chromatography, silica gel (Sigma Aldrich 60 mesh) was used. Unless otherwise stated, HPLC purifications were performed on a semi-preparative HPLC column (Waters XBridge Prep C18 5 μM, 19 × 100 mm) connected to an Agilent (Santa Clara, CA, USA) 1260 Infinity LC system. Pre-coated silica gel plates (Chemscene HPTLC Silica Gel 60 F254) were used for thin-layer chromatography. The microwave used for deprotection and cyclization was the Biotage (Salem, NH, USA) Initiator.

### 4.2. Purification and Isolation of Natural 2,5-diketopiperazine

Thirty liters of Wheatgrass MMC was extracted with ethyl acetate to yield 800 mg of crude material. This was fractionated on three silica gel columns with dichloromethane/methanol gradients. Fraction 2-5-13 (8 mg) was subjected to analytical HPLC (settings: Agilent Zorbax 5 µm C8 column, mobile phase 30–100% methanol, 0–5 min 0–30% ramp, ~21 min 100%, total run time 30 min, flow rate 1 mL/min). UV-active (254 nm) fractions were collected. They were screened with LC-MS and the ten-minute fraction was found to contain pure material. Samples were dried in vacuo to afford 0.3 mg of purified material. MS analysis suggested the presence of two isomers of cyclo(Leu-Pro) in the fraction collected between five and ten minutes, but the amounts were too small to isolate. 

### 4.3. Construction of Synthetic DKP Library

2,5-diketopiperazines (DKP) were synthesized as described previously with a minor modification [16]: the microwave temperature used was 180 °C instead of 200 °C. 

### 4.4. Purification of Synthetic DKPs

Samples were eluted from the silica gel column with 95% dichloromethane:5% methanol. Further purification was carried out with semi-preparative HPLC (a linear gradient of 5–95% acetonitrile in water with 0.05% TFA over 10 min, flow rate 15 mL/min with 210 and 254 nm detection). Fractions containing cyclo(Phe-Pro) eluted between 7 and 7.6 min (254 nm), cyclo(Leu-Pro) eluted between 6.4 and 6.7 min (210 nm), and cyclo(Val-Pro) eluted between 4.4 and 4.7 min (210 nm). 

*Cyclo(L-Phe-L-Pro)/(3S,8aS)-3-benzylhexahydropyrrolo[1,2-a]pyrazine-1,4-dione **1***

^1^H NMR (500 MHz, CDCl_3_) δ 7.38 (t, *J* = 7.3 Hz, 2H), 7.31 (t, *J* = 7.2 Hz, 1H), 7.25 (d, *J* = 7.5 Hz, 2H), 5.92 (s, 1H), 4.32 (dd, *J* = 10.4, 3.7 Hz, 1H), 4.11 (t, *J* = 8.1 Hz, 1H), 3.68 (dt, *J* = 12.0, 7.9 Hz, 1H), 3.65–3.54 (m, 2H), 2.84 (dd, *J* = 14.5, 10.3 Hz, 1H), 2.36 (dt, *J* = 13.4, 5.7 Hz, 1H), 2.08–2.02 (m, 1H), 2.02–1.87 (m, 2H).^13^C NMR (126 MHz, CDCl_3_) δ 169.70, 165.05, 135.77, 129.27, 129.17, 127.60, 59.12, 56.31, 45.49, 36.80, 28.33, 22.51. UV (*methanol*): (*ε*) = 220 nm (3700); HRMS (ESI, *m*/*z*): [M + H]^+^ calculated for C_14_H_16_N_2_O_2_, 245.1285; observed, 245.1290.

*Cyclo(L-Phe-D-Pro)/(3S,8aR)-3-benzylhexahydropyrrolo[1,2-a]pyrazine-1,4-dione **2***

^1^H NMR (500 MHz, CDCl_3_) δ 7.39 (d, *J* = 3.7 Hz, 1H), 7.31 (s, 2H), 7.21 (d, *J* = 6.6 Hz, 2H), 4.31 (q, *J* = 4.6 Hz, 1H), 3.61 (dt, *J* = 11.6, 8.5 Hz, 1H), 3.43–3.31 (m, 1H), 3.21 (dd, *J* = 13.8, 5.6 Hz, 1H), 3.06 (dd, *J* = 13.8, 4.2 Hz, 1H), 2.75 (dd, *J* = 10.8, 6.4 Hz, 1H), 2.15 (dt, *J* = 12.4, 6.4 Hz, 1H), 1.96–1.86 (m, 1H), 1.79–1.62 (m, 2H). ^13^C NMR (126 MHz, CDCl_3_) δ 170.03, 164.14, 134.79, 130.00, 128.71, 127.31, 58.34, 56.99, 44.35, 39.16, 28.74, 21.02. UV (*methanol*): (*ε*) = 220 nm (4800); HRMS (ESI, *m*/*z*): [M + H]^+^ calculated for C_14_H_16_N_2_O_2_, 245.1285; observed, 245.1287. 

*Cyclo(D-Phe-L-Pro)/(3R,8aS)-3-benzylhexahydropyrrolo[1,2-a]pyrazine-1,4-dione **3***

^1^H NMR (500 MHz, CDCl_3_) δ 7.41 (d, *J* = 3.7 Hz, 1H), 7.31 (d, *J* = 2.1 Hz, 2H), 7.21 (dd, *J* = 6.8, 2.5 Hz, 2H), 4.31 (q, *J* = 4.6 Hz, 1H), 3.61 (dt, *J* = 12.2, 8.5 Hz, 1H), 3.42–3.34 (m, 1H), 3.21 (dd, *J* = 13.7, 5.7 Hz, 1H), 3.06 (dd, *J* = 13.7, 4.3 Hz, 1H), 2.75 (dd, *J* = 10.7, 6.4 Hz, 1H), 2.15 (dq, *J* = 12.2, 6.0, 5.3 Hz, 1H), 1.98–1.90 (m, 1H), 1.79–1.64 (m, 2H). ^13^C NMR (126 MHz, CDCl_3_) δ 170.06, 165.12, 135.01, 130.00, 128.72, 127.60, 58.70, 57.67, 45.15, 40.28, 28.82, 21.56. UV (*methanol*): (*ε*) = 220 nm (4800); HRMS (ESI, *m*/*z*): [M + H]^+^ calculated for C_14_H_16_N_2_O_2_, 245.1285; observed, 245.1288.

*Cyclo(D-Phe-D-Pro)/(3R,8aR)-3-benzylhexahydropyrrolo[1,2-a]pyrazine-1,4-dione **4***

^1^H NMR (500 MHz, CDCl_3_) δ 7.37 (t, *J* = 7.4 Hz, 2H), 7.31 (t, *J* = 7.3 Hz, 1H), 7.27–7.23 (m, 2H), 6.07 (s, 1H), 4.36–4.27 (m, 1H), 4.11 (t, *J* = 6.2, 4.0 Hz, 1H), 3.68 (dt, *J* = 11.9, 7.8 Hz, 1H), 3.64–3.55 (m, 2H), 2.86 (dd, *J* = 14.5, 10.2 Hz, 1H), 2.40–2.29 (m, 1H), 2.09–1.99 (m, 1H), 2.00–1.86 (m, 2H). ^13^C NMR (126 MHz, CDCl_3_) δ 169.86, 165.05, 135.68, 129.26, 129.21, 127.62, 77.29, 77.03, 76.78, 59.11, 56.36, 45.51, 37.94, 36.79, 28.32, 22.48. UV (*methanol*): (*ε*) = 220 nm (3700); HRMS (ESI, *m*/*z*): [M + H]^+^ calculated for C_14_H_16_N_2_O_2_, 245.1285; observed, 245.1290.

*Cyclo(L-Leu-L-Pro)/(3S,8aS)-3-isobutylhexahydropyrrolo[1,2-a]pyrazine-1,4-dione **5***

^1^H NMR (500 MHz, CDCl_3_) δ 5.92 (s, 1H), 4.12 (dd, *J* = 9.2, 7.3 Hz, 1H), 4.02 (dd, *J* = 10.0, 3.8 Hz, 1H), 3.65–3.50 (m, 2H), 2.40–2.30 (m, 1H), 2.18–2.10 (m, 1H), 2.09–1.99 (m, 2H), 1.96–1.83 (m, 1H), 1.82–1.68 (m, 1H), 1.57–1.48 (m, 1H), 1.00 (d, *J* = 6.6 Hz, 3H), 0.95 (d, *J* = 6.6 Hz, 3H). ^13^C NMR (126 MHz, CDCl_3_) δ 170.17, 166.15, 59.00, 53.40, 45.52, 38.64, 28.13, 24.73, 23.30, 22.75, 21.20. UV (*methanol*): (*ε*) = 220 nm (1800); HRMS (ESI, *m*/*z*): [M + H]^+^ calculated for C_11_H_18_N_2_O_2_, 211.1441; observed, 211.1442.

*Cyclo(L-Leu-D-Pro)/(3S,8aR)-3-isobutylhexahydropyrrolo[1,2-a]pyrazine-1,4-dione **6***

^1^H NMR (500 MHz, CDCl_3_) δ 6.93 (s, 1H), 4.10 (dd, *J* = 9.7, 6.5 Hz, 1H), 3.99–3.91 (m, 1H), 3.64 (d, *J* = 2.3 Hz, 1H), 3.55 (dd, *J* = 9.1, 2.4 Hz, 1H), 2.44–2.36 (m, 1H), 2.09–1.97 (m, 2H), 1.95–1.86 (m, 1H), 1.82–1.74 (m, 1H), 1.72–1.57 (m, 2H), 0.97 (dd, *J* = 17.6, 6.6 Hz, 6H). ^13^C NMR (126 MHz, CDCl_3_) δ 169.76, 166.45, 58.03, 56.25, 45.66, 42.50, 28.93, 24.43, 23.04, 22.20, 21.31. UV (*methanol*): (*ε*) = 220 nm (1800); HRMS (ESI, *m*/*z*): [M + H]^+^ calculated for C_11_H_18_N_2_O_2_, 211.1441; observed, 211.1439.

*Cyclo(D-Leu-L-Pro)/(3R,8aS)-3-isobutylhexahydropyrrolo[1,2-a]pyrazine-1,4-dione **7***

^1^H NMR (500 MHz, CDCl_3_) δ 7.37 (s, 1H), 4.10 (dd, *J* = 9.9, 6.5 Hz, 1H), 3.97 (dt, *J* = 9.7, 5.2 Hz, 1H), 3.69–3.59 (m, 1H), 3.59–3.48 (m, 1H), 2.48–2.31 (m, 1H), 2.12–1.95 (m, 2H), 1.90 (dd, *J* = 9.8, 7.0 Hz, 1H), 1.84–1.73 (m, 1H), 1.72–1.54 (m, 2H), 0.99 (d, *J* = 6.6 Hz, 3H), 0.96 (d, *J* = 6.5 Hz, 3H). ^13^C NMR (126 MHz, CDCl_3_) δ 170.00, 166.63, 58.05, 56.18, 45.69, 42.49, 28.91, 24.43, 23.04, 22.19, 21.31. UV (*methanol*): (*ε*) = 220 nm (1800); HRMS (ESI, *m*/*z*): [M + H]^+^ calculated for C_11_H_18_N_2_O_2_, 211.1441; observed, 211.1444.

*Cyclo(D-Leu-D-Pro)/(3R,8aR)-3-isobutylhexahydropyrrolo[1,2-a]pyrazine-1,4-dione **8***

^1^H NMR (500 MHz, CDCl_3_) δ 5.92 (s, 1H), 4.12 (dd, *J* = 9.2, 7.4 Hz, 1H), 4.02 (dd, *J* = 9.6, 3.8 Hz, 1H), 3.65–3.50 (m, 2H), 2.40–2.31 (m, 1H), 2.19–2.11 (m, 1H), 2.11–1.97 (m, 2H), 1.97–1.84 (m, 1H), 1.81–1.69 (m, 2H), 1.57–1.48 (m, 1H), 1.00 (d, *J* = 6.6 Hz, 3H), 0.96 (d, *J* = 6.5 Hz, 3H). ^13^C NMR (126 MHz, CDCl_3_) δ 170.15, 166.16, 59.00, 53.40, 45.52, 38.63, 28.12, 24.73, 23.30, 22.75, 21.20. UV (*methanol*): (*ε*) = 220 nm (1800); HRMS (ESI, *m*/*z*): [M + H]^+^ calculated for C_11_H_18_N_2_O_2_, 211.1441; observed, 211.1442.

*Cyclo(L-Val-L-Pro)/(3S,8aS)-3-isopropylhexahydropyrrolo[1,2-a]pyrazine-1,4-dione **9***

^1^H NMR (500 MHz, CDCl_3_) δ 6.01 (s, 1H), 4.09 (t, *J* = 8.2 Hz, 1H), 3.95 (d, *J* = 2.2 Hz, 1H), 3.68–3.61 (m, 1H), 3.58–3.49 (m, 1H), 2.68–2.61 (m, 1H), 2.39 (dt, *J* = 12.4, 7.1 Hz, 1H), 2.10–1.99 (m, 2H), 1.96–1.88 (m, 1H), 1.07 (d, *J* = 7.2 Hz, 3H), 0.91 (d, *J* = 6.7 Hz, 3H). ^13^C NMR (126 MHz, CDCl_3_) δ 170.13, 164.89, 60.39, 58.83, 45.18, 28.54, 28.35, 22.39, 19.29, 16.06. UV (*methanol*): (*ε*) = 220 nm (1600); HRMS (ESI, *m*/*z*): [M + H]^+^ calculated for C_10_H_16_N_2_O_2_, 197.1285; observed, 197.1287.

*Cyclo(L-Val-D-Pro)/(3S,8aR)-3-isopropylhexahydropyrrolo[1,2-a]pyrazine-1,4-dione **10***

^1^H NMR (500 MHz, CDCl_3_) δ 7.17 (s, 1H), 4.10 (dd, *J* = 10.1, 6.5 Hz, 1H), 3.76 (q, *J* = 4.7, 3.5 Hz, 1H), 3.74–3.67 (m, 1H), 3.57–3.49 (m, 1H), 2.46–2.37 (m, 1H), 2.24 (q, *J* = 7.1 Hz, 1H), 2.05 (dq, *J* = 8.4, 2.7 Hz, 1H), 1.99–1.85 (m, 2H), 1.05 (d, *J* = 7.0 Hz, 3H), 1.00 (d, *J* = 6.9, 3H). ^13^C NMR (126 MHz, CDCl_3_) δ 169.77, 165.39, 63.44, 58.32, 45.59, 33.15, 29.39, 21.93, 18.99, 17.55. UV (*methanol*): (*ε*) = 220 nm (1700); HRMS (ESI, *m*/*z*): [M + H]^+^ calculated for C_10_H_16_N_2_O_2_, 197.1285; observed, 197.1286.

*Cyclo(D-Val-L-Pro)/(3R,8aS)-3-isopropylhexahydropyrrolo[1,2-a]pyrazine-1,4-dione **11***

^1^H NMR (500 MHz, CDCl_3_) δ 7.58 (s, 1H), 4.12 (dd, *J* = 10.2, 6.3 Hz, 1H), 3.83–3.75 (m, 1H), 3.70 (dt, *J* = 12.3, 8.5 Hz, 1H), 3.59–3.49 (m, 1H), 2.41 (dt, *J* = 11.3, 6.0 Hz, 1H), 2.29–2.18 (m, 1H), 2.09–2.02 (m, 1H), 1.99–1.83 (m, 2H), 1.05 (d, *J* = 6.7 Hz, 3H), 1.00 (d, *J* = 6.6 Hz, 3H). ^13^C NMR (126 MHz, CDCl_3_) δ 170.09, 165.58, 63.34, 58.34, 45.64, 33.18, 29.36, 21.92, 18.97, 17.57. UV (*methanol*): (*ε*) = 220 nm (1700); HRMS (ESI, *m*/*z*): [M + H]^+^ calculated for C_10_H_16_N_2_O_2_, 197.1285; observed, 197.1303.

*Cyclo(D-Val-D-Pro)/(3R,8aR)-3-isopropylhexahydropyrrolo[1,2-a]pyrazine-1,4-dione **12***

^1^H NMR (500 MHz, CDCl_3_) δ 5.97 (s, 1H), 4.08 (t, *J* = 8.3 Hz, 1H), 3.95 (d, *J* = 2.1 Hz, 1H), 3.64 (dt, *J* = 12.3, 8.1 Hz, 1H), 3.59–3.48 (m, 1H), 2.70–2.59 (m, 1H), 2.43–2.33 (m, 1H), 2.17–1.98 (m, 2H), 1.97–1.84 (m, 1H), 1.07 (d, *J* = 7.3, 3H), 0.91 (d, *J* = 6.8, 3H). ^13^C NMR (126 MHz, CDCl_3_) δ 170.08, 164.86, 60.37, 58.81, 45.15, 28.52, 28.32, 22.37, 19.28, 16.03. UV (*methanol*): (*ε*) = 220 nm (1600); HRMS (ESI, *m*/*z*): [M + H]^+^ calculated for C_10_H_16_N_2_O_2_, 197.1285; observed, 197.1289.

### 4.5. E. coli Growth and Biofilm Assays

A static biofilm formation assay was performed in a 96-well polystyrene microtiter plate, as described previously with minor modifications [30]. An overnight culture of *E. coli* (BL21(DE3)) was diluted 1:10, reaching a ~0.2 OD_600_. Then, 10 mg/mL stock solutions of purified synthetic DKPs were prepared in dimethyl sulfoxide, and 0.1 mg/mL of the DKPs were added to 200 μL of bacterial cell culture. The plates were kept at a stationary state at 37 °C for 24 h. The growth of *E. coli* in each well was first estimated by the OD_600_ measurement. Then, wells were washed twice with deionized water gently to remove the planktonic cells, and the cells were then incubated for 30 min at 60 °C to fix biofilm. The cells in the biofilm were stained with 0.2% crystal violet solution for 15 min. Unbound dye in the wells was washed off with deionized water. The excess water was left to dry at room temperature for one hour, then the CV was dissolved in 33% acetic acid, and absorbances were measured at 595 nm (OD_595_) to quantify biofilm formation. To ensure the reproducibility, a total of 50 replicates were examined for each treatment.

## 5. Conclusions

Studies on the stereochemistry of DKPs and their biological significance have long been hampered by the lack of suitable methods for their stereochemical assignment. Here, we demonstrated that ECD can serve as a simple, sensitive, and reproducible method for the stereochemical characterization of DKPs. Furthermore, the synthetic DKP library from this study has created a new opportunity to start exploring the stereochemistry–activity relationship of this ubiquitous but poorly characterized group of microbial metabolites. 

## Figures and Tables

**Table 1:** UV *ε*-values of DKP stereoisomers in methanol (spectroscopic grade).

Compounds	*ε*-value (220 nm)
**1**, **4**	3700
**2**, **3**	4800
**5**, **8**	1800
**6**, **7**	1800
**9**, **12**	1600
**10**, **11**	1700

## Data Availability

Mass spectrometric datasets are available from GNPS under accession number MSV000086394.

## Supplementary Materials

The following are available online, Figure S1: GNPS analysis of metabolites in wheatgrass MMC.

## Sample Availability

Samples of the synthetic DKPs **1**–**12** are available from the authors upon request.
