# Supplementary material for: Unambiguous Stereochemical Assignment of Cyclo(Phe-Pro), Cyclo(Leu-Pro), and Cyclo(Val-Pro) by Electronic Circular Dichroic Spectroscopy
Source: Molecules. 2021 Oct 2;26(19):5981. doi: 10.3390/molecules26195981 (PMC8512403; doi:10.3390/molecules26195981)
Supplement: Supplementary file 1 [file molecules-26-05981-s001.zip › molecules-1367872-SI.pdf]

Unambiguous stereochemical assignment of cyclo(Phe-Pro), cyclo(Leu-Pro), and cyclo(Val-Pro) by electronic circular dichroic spectroscopy

Alison Domzalski, Liliana Margent, Valeria Vigo, Faizunnahar Dewan, Nava Gara Kishore Pilarsetty, Yujia Xu, and Akira Kawamura

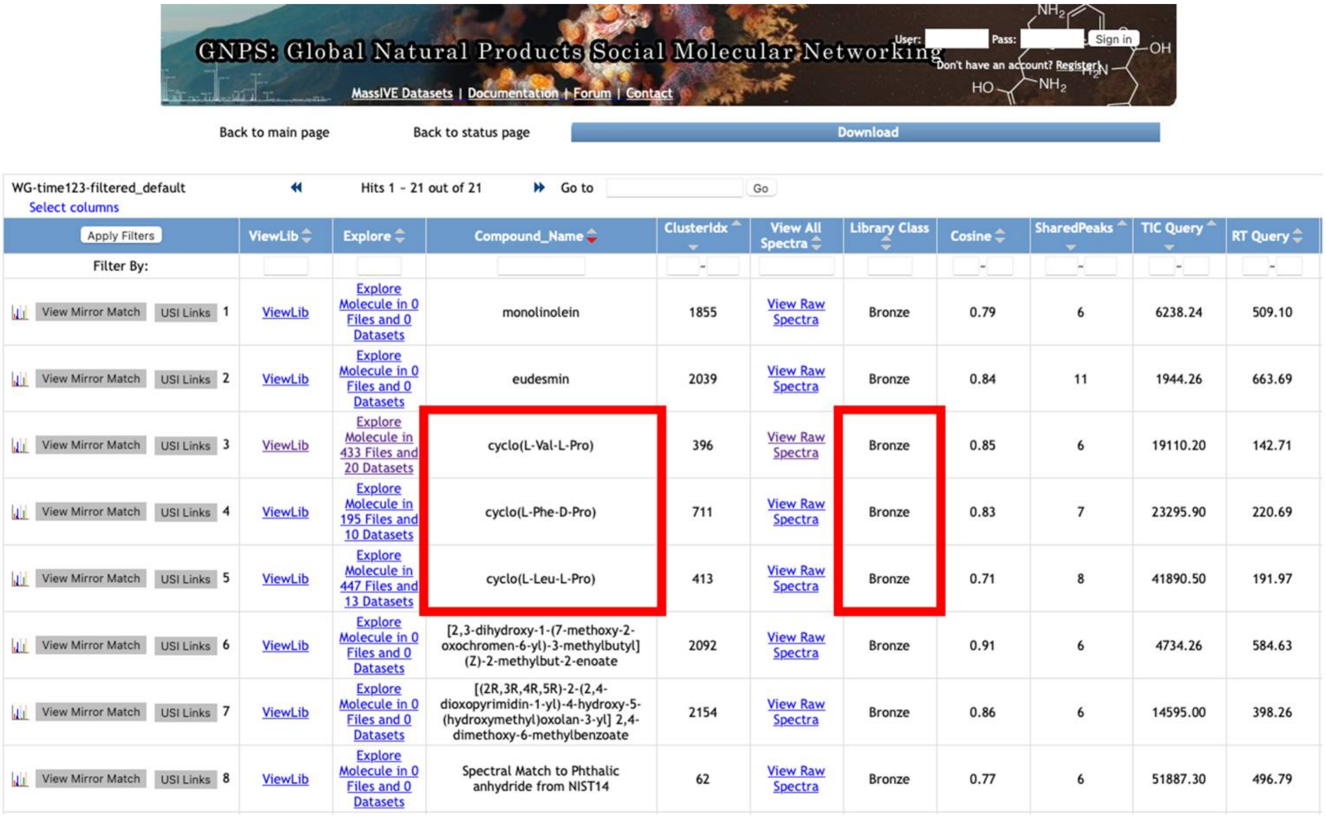

#	ViewLib	Explore	Compound_Name	ClusterIdx	View All Spectra	Library Class	Cosine	SharedPeaks	TIC Query	RT Query
View Mirror Match USI Links 1	ViewLib	Explore Molecule in 0 Files and 0 Datasets	monolinolein	1855	View Raw Spectra	Bronze	0.79	6	6238.24	509.10
View Mirror Match USI Links 2	ViewLib	Explore Molecule in 0 Files and 0 Datasets	eudesmin	2039	View Raw Spectra	Bronze	0.84	11	1944.26	663.69
View Mirror Match USI Links 3	ViewLib	Explore Molecule in 433 Files and 20 Datasets	cyclo(L-Val-L-Pro)	396	View Raw Spectra	Bronze	0.85	6	19110.20	142.71
View Mirror Match USI Links 4	ViewLib	Explore Molecule in 195 Files and 10 Datasets	cyclo(L-Phe-D-Pro)	711	View Raw Spectra	Bronze	0.83	7	23295.90	220.69
View Mirror Match USI Links 5	ViewLib	Explore Molecule in 447 Files and 13 Datasets	cyclo(L-Leu-L-Pro)	413	View Raw Spectra	Bronze	0.71	8	41890.50	191.97
View Mirror Match USI Links 6	ViewLib	Explore Molecule in 0 Files and 0 Datasets	[2,3-dihydroxy-1-(7-methoxy-2-oxochromen-6-yl)-3-methylbutyl] (Z)-2-methylbut-2-enoate	2092	View Raw Spectra	Bronze	0.91	6	4734.26	584.63
View Mirror Match USI Links 7	ViewLib	Explore Molecule in 0 Files and 0 Datasets	[(2R,3R,4R,5R)-2-(2,4-dioxopyrimidin-1-yl)-4-hydroxy-5-(hydroxymethyl)oxolan-3-yl] 2,4-dimethoxy-6-methylbenzoate	2154	View Raw Spectra	Bronze	0.86	6	14595.00	398.26
View Mirror Match USI Links 8	ViewLib	Explore Molecule in 0 Files and 0 Datasets	Spectral Match to Phthalic anhydride from NIST14	62	View Raw Spectra	Bronze	0.77	6	51887.30	496.79

Figure S1. GNPS analysis of metabolites in wheatgrass MMC. A LC/MS/MS dataset of wheatgrass MMC extract was submitted to GNPS, which identified a series of metabolites, including cyclo(L-Val-L-Pro), cyclo(L-Phe-D-Pro), and cyclo(L-Leu-L-Pro) as "Bronze" matches. These DKPs were also observed in the GNPS analyses of other MMCs (not shown).
